# CCL2/CCL5 secreted by the stroma induce IL-6/PYK2 dependent chemoresistance in ovarian cancer

**DOI:** 10.1186/s12943-018-0787-z

**Published:** 2018-02-19

**Authors:** Jennifer Pasquier, Marie Gosset, Caroline Geyl, Jessica Hoarau-Véchot, Audrey Chevrot, Marc Pocard, Massoud Mirshahi, Raphael Lis, Arash Rafii, Cyril Touboul

**Affiliations:** 1 Stem cell and microenvironment laboratory, Weill Cornell Medical College in Qatar, Education City, Qatar Foundation, Doha, Qatar; 2 Department Genetic Medicine, Weill Cornell Medical College, New York, NY USA; 3 INSERM U955, Equipe 7, Créteil, France; 4 UMR INSERM U965: Angiogenèse et Recherche translationnelle. Hôpital Lariboisière, 49 bd de la chapelle, 75010 Paris, France; 5 Faculté de médecine de Créteil UPEC – Paris XII. Service de Gynécologie-Obstétrique et Médecine de la Reproduction. Centre Hospitalier Intercommunal de Créteil, 40 Avenue de Verdun, 94000 Créteil, France

**Keywords:** Ovarian cancer, Chemoresistance, Il-6, Mesenchymal stromal cell, Mouse

## Abstract

**Background:**

Minimal residual disease is the main issue of advanced ovarian cancer treatment. According to the literature and previous results, we hypothesized that Mesenchymal Stromal Cells (MSC) could support this minimal residual disease by protecting ovarian cancer cells (OCC) from chemotherapy*.* In vitro study confirmed that MSC could induce OCC chemoresistance without contact using transwell setting. Further experiments showed that this induced chemoresistance was dependent on IL-6 OCC stimulation.

**Methods:**

We combined meticulous in vitro profiling and tumor xenograft models to study the role of IL-6 in MSC/OCC intereactions.

**Results:**

We demonstrated that Tocilizumab® (anti-IL-6R therapy) in association with chemotherapy significantly reduced the peritoneal carcinosis index (PCI) than chemotherapy alone in mice xenografted with OCCs+MSCs. Further experiments showed that CCL2 and CCL5 are released by MSC in transwell co-culture and induce OCCs IL-6 secretion and chemoresistance. Finally, we found that IL-6 induced chemoresistance was dependent on PYK2 phosphorylation.

**Conclusions:**

These findings highlight the potential key role of the stroma in protecting minimal residual disease from chemotherapy, thus favoring recurrences. Future clinical trials targeting stroma could use anti-IL-6 therapy in association with chemotherapy.

**Electronic supplementary material:**

The online version of this article (10.1186/s12943-018-0787-z) contains supplementary material, which is available to authorized users.

## Background

Ovarian cancer remains a challenging condition for both clinicians and scientist as it is the deadliest gynecologic cancer [1]. While most of the patient will respond to the chemotherapy, 75% will die of their disease with a high rate of recurrence within the first 2 years (40 to 50%). It often presents as an advanced disease with peritoneal carcinosis however most patients are treated with a combination of major debulking surgeries and platinium based chemotherapy to achieve complete cytoreduction, i.e. no visible residual disease. The clinical course of patients with no residue at the end of the treatment remains unpredictable with a group of early recurrence (refractory patients). The clinical trials of targeted therapies such as trastuzumab, imatinib, or bevacizumab, as well as dose intensifications or use of several agents have experience difficulties in improving overall survival [2–4]. Recently, poly-ADP-ribose-polymerase (PARP) inhibitors showed a significant survival improvement in patients BRCA mutated or with DNA homologous repair deficiency (HRD) [5].

One area that has gained tremendous interest over the last decade is the role of the microenvironment in the biology of neoplastic diseases [6]. Several studies have illustrated the crucial role of the cellular elements of the tumor stroma: cancer associated fibroblasts [7], tumor associated macrophage [8], mesenchymal stromal cells [9–14], or endothelial cells [4, 15, 16]. Among these elements, mesenchymal Stromal Cells (MSC) have been widely studied and shown to be essential during the invasion of the stroma by ovarian cancer cells (OCCs), after dissociation of the mesothelial layer [17]. MSCs are pluripotent stromal cells that give rise to a variety of connective tissues – adipose, bone, cartilage and muscle – and secrete specific cytokines and growth factors [18]. Based on the tropism of MSCs for the tumor microenvironment, numerous studies have suggested that MSCs could be potentially targeted during therapeutic treatment of tumors [9–12, 19]. In order to achieve such ambitious goal, one should understand precisely the role of MSC in cancer progression.

Our previous studies had shown that OCCs in contact with MSCs exhibit a pro-metastatic and chemoresistant profile [9, 11, 12]. We described the key role of IL-6 in the interaction between these two cell types [12]. To further clarify the role of IL-6 and the secreted factors, we designed an experimental model in which mesenchymal cells extracted from neoplastic ascites interact, in a serum-free environment, with ovarian cancer cells exclusively through secreted factors.

Combining meticulous molecular profiling and tumor xenograft models, we demonstrated that Tocilizumab® (anti-IL6R therapy) in association with chemotherapy significantly reduced the peritoneal carcinosis index (PCI) compared to chemotherapy alone in mice xenografted with OCCs+MSCs. We demonstrated that OCC co-opt MSC’s secretion of CCL2 and CCL5 resulting in activation of an autocrine loop in OCCs and subsequently resistance to therapy. Finally, we found that IL6 induced chemoresistance was dependent on PYK2 phosphorylation.

## Methods

### Cell culture

#### Ovarian cancer cell

Ovarian cancer cell lines Skov3 and Ovcar3 were purchased from ATCC and cultured following ATCC recommendations (ATCC, Manassas, VA, USA). A primary ovarian cancer cell line was derived in our laboratory from ascites of a patient with Stage III serous adenocarcinoma (APOCC) [20]. The 3 cell lines were cultured in DMEM high glucose (Hyclone, Thermo Scientific), 10% FBS (Hyclone, Thermo Scientific), 1% Penicillin-Streptomycin-Amphotericyn B solution (Sigma), 1X Non-Essential Amino-Acid (Hyclone, Thermo Scientific) and 1% L-glutamine. Cultures were incubated in humidified 5% CO2 incubators at 37 °C, media was replaced every 3 days.

#### Mesenchymal cells

We isolated mesenchymal cells from ascites of a patient with Stage III serous adenocarcinoma (Additional file 1: Figure S1A). Ascites fluid was centrifuged and the pelleted cells were plated on plastic in DMEM low glucose [Hyclone, Thermo Scientific], 20% FBS [Hyclone, Thermo Scientific], 1% Penicillin-Streptomycin-Amphotericyn B solution [Sigma]. After one week, EpCAM^−^ cells were sorted and cultured on plastic for 3 passages. Sorted cells have morphology of MSC with long thin cell bodies with a large nucleus (Additional file 1: Figure S1B). In order to confirm this first observation, we performed a phenotypic analysis by flow cytometry using MSC markers (Additional file 1: Figure S1C). The cells have a MSC phenotype: Lin^−^, CD45^−^, CD73^+^, CD105^+^, CD29^+^, and CD90^+^. Finally, we confirmed the positive markers by immunostaining by confocal microscopy (Additional file 1: Figure S1D).

### Enzyme-linked immunosorbent assay (ELISA)

For ELISA we used a Human IL-6 Quantikine ELISA Kit from R&D systems (#S6050). ELISA was performed on cell supernatants according to the manufacturer protocol. PYK2 inhibitor (PF 431396) had been purchased in Tocris (Cat. No. 4278).

### Confocal microscopy

Imaging was performed using a Zeiss confocal Laser Scanning Microscope 710 (Carl Zeiss) as previously described [14]. Post-acquisition image analysis was performed with Zeiss LSM Image Browser Version 4.2.0.121 (Carl Zeiss).

#### Calcein-AM staining

For the calcein-AM assay, cells were prepared as previously described [10]. Briefly, cells were stained with 0,25 *µ*M of calcein-AM. After 15 min incubation at 37 °C, cells were washed twice with PBS.

### Flow cytometry

Fluorescence (FL) was quantified on a SORP FACSAria2 (BD Biosciences) as previously described [14, 16]. Data were processed with FACSDiva 6.3 software (BD Biosciences). Doublets were excluded by FSC-W x FSC-H and SSC-W x SSC-H analysis. Charts display the median of fluorescence intensity (mfi) relative to control. Single stained channels were used for compensation and fluorophore minus one (FMO) controls were used for gating. 20,000 events were acquired per sample.

Cells from ascites fluids were stained with EpCam APC conjugated (BD Biosciences) and the fluorescence was acquired with 647 nm red laser and 670/14 nm emission.

MSC were defined as Lin^−^CD45^−^CD90^+^CD73^+^CD105^+^CD29^+^. The cell suspension was stained with mouse anti-human CD45 antibody (BD biosciences, #339192, clone 2D1) coupled with Amcyan, anti-mouse lineage cocktail 1 (Lin, BD biosciences, #340546, CD3, CD14, CD16, CD19, CD20, CD56) coupled with FITC, mouse anti-human CD105 (biolegend, #323212, clone 43A3) coupled with AF647, mouse anti-human CD73 (BD biosciences, #550257, clone AD2) coupled with PE, mouse anti-human CD29 (biolegend, #323212, clone TS2/16) coupled with APC-Cy7, mouse anti-human CD90 (BD biosciences, #550402, clone 5E10) coupled with AF700.

### Western blot analysis

Western blot were carried out as previously described [14]. Immunostaining was carried out using a goat monoclonal antibody against Phospho PYK2 #3291, PYK2 #3292, IL-6 #2153, actin #3200 (1/1000, Cell signaling) and a secondary polyclonal mouse anti-goat antibody HRP conjugated (1/2000, cell signaling). Blots were developed using HRP and chemiluminescent peroxidase substrate (#CPS1120, Sigma). Data were collected using Geliance CCD camera (Perkin Elmer), and analyzed using ImageJ software (NIH).

### Cytokine array

All cells were starved for 24 h prior the cytokine Array experiment. 200 μg of protein was loaded on RayBio® Human Cytokine Antibody Array G Series 1000 (Raybiotec, Norcross, GA) according to manufacturer’s instructions. Arrays were revealed using HorseRadish Peroxidase (HRP) and SuperSignal West Pico Chemiluminescent Substrate (Thermo-Scientific, Dubai, Emirates). Data were collected using Geliance CCD camera (Perkin Elmer, MA), and extracted using ImageJ software (NIH). Briefly, the pictures of the arrays were inverted and background subtracted. We then defined the area for signal capture for all spots as 110–120 μm diameter, using the same area for every spot. We defined our signal as the median pixel density value. For the comparison, the independent arrays values were normalized on their positive control intensity value.

### Animal study

#### Study groups

Nude mice were obtained from Charles River (4 weeks NU/NU Nude mouse). Animals were maintained in accordance with institutional policies, and all studies were performed with approval of the University Committee on Use and Care of Animals of the University of Paris V – Diderot, France (n°02095.03). Five groups of mice were studied to investigate the impact of Tocilizumab®, an anti-IL6R therapy, in association with chemotherapy on the ovarian peritoneal carcinosis. Assuming a mean difference of peritoneal carcinosis index of 8 (e.g. 15 to 7) with tocilizumab® in addition to chemotherapy with a risk α= 0.05 and β=80%, we needed 7 mice per group. To be sure that we could show a difference, we considered 8 mice per test group as adequate. To generate peritoneal carcinosis, 6 × 10^6^ Ovcar3 cells in 5 mL of medium without FBS were injected intra-peritoneally in nude mice or a coinjection of 2:1 mixture of 4 × 10^6^ Ovcar3 cells with 2 × 10^6^ MSCs 6 × 10^6^ Ovcar3 cells in order to investigate the impact of the microenvironment. All studies were done using early-passage amniotic membrane MSCs (passage 3–8). Peritoneal carcinosis was monitored using bioluminescence. Two control groups without treatment and three groups with chemotherapy +/− Tocilizumab® were studied.

#### Tumor imaging

Ovcar3 tumor cells were stably transduced with a luciferase-expressing lentivirus (plentiloxEV-Luc virus, provided by the University of Toulouse). Bioluminescence optical imaging (Xenogen IVIS 2000, Caliper Life Sciences) was obtained 7 and 14 days after tumor cell injection. Ten minutes prior to imaging, each mouse was given an i.p. injection with 100 μl coelenterazine in PBS at 40 mg/ml. During the imaging, general anesthesia was given with 2% isoflurane (IsoSol, Medeva Pharmaceuticals Inc.). Luminescence images were acquired for 3 s to 1 min. The optical signal was expressed as radiance in units of photons/s/cm2 (p/s/cm2). We excluded the mice from the analysis when no signal was observed, meaning a failure of the xenograft.

#### Treatment

Three weeks after xenograft, the mice received intra-peritoneal injections of Carboplatin® twice a week of 10,76 mM of carboplatin in 200 μL 5% glucose solution +/− intra-peritoneal injections 3× per week of 10,76 mM Tocilizumab® at the dose of 5 mg/kg, i.e. 125 μg in 100 μL saline solution.

#### Carcinosis evaluation

The tumor burden was monitored by bioluminescence during the treatment. We evaluated the peritoneal index after sacrifice of the mice at the end of the 3 weeks treatment. We used the peritoneal carcinosis index modified for mice (cf dohan, lousquy, am j pathol 2014 ou 15) and the use of bioluminescence allowed to increase the identification of small nodules difficult to detect by naked eyes.

### RT-PCR analysis

Total RNA was extracted from cells cultures using Trizol. After genomic DNA removal by DNase digestion (Turbo DNA free kit, Applied Biosystems), total RNA (1 μg) was reverse transcribed with oligodT (Promega) using the Superscript III First-Strand Synthesis SuperMix (Invitrogen). PCR analysis was performed with a MasterCycler apparatus (Eppendorf) from 2 μL of cDNA using primers from IDT (Additional file 2: Table S1).

### Statistical analysis

All quantitative data were expressed as mean ± standard error of the mean (SEM). Statistical analysis was performed with SigmaPlot 11 (Systat Software Inc., Chicago, IL). A Shapiro-Wilk normality test, with a *p* = 0.05 rejection value, was used to test normal distribution of data prior further analysis. All pairwise multiple comparisons were performed by one way ANOVA followed by Holm-Sidak posthoc tests for data with normal distribution or by Kruskal-Wallis analysis of variance on ranks followed by Tukey posthoc tests, in case of failed normality test. Paired comparisons were performed by Student’s t-tests or by Mann-Whitney rank sum tests in case of unequal variance or failed normality test. All experiments were performed in triplicates. We used Wilcoxon test to compare mean peritoneal carcinosis index between the different treatment regimen groups of mice. Statistical significance was accepted for *p* < 0.05 (*), *p* < 0.01 (**) or *p* < 0.001 (***).

## Results

### MSC cells protect OCCs from chemotherapy without contact through IL-6

**Fig. 1:**
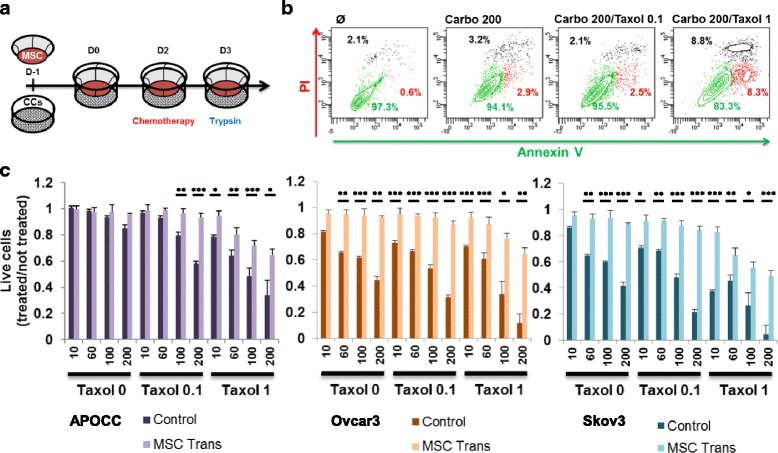
**a**. At D-1, MSC were plated on a transwell and ovarian cancer cells (OCCs) on a well itself. At D0, MSC and OCCs were put in contact. At D2 the chemotherapy were added for 24 h. At D3, cells were harvested for analysis. **b**. MSC were treated with Carboplatin (200 μM) or with a combination of Carboplatin (200 μM) and Taxol (0.1 μM or 1 μM) for 24 h. MSC were stained with PI and Annexin V, percentage of live cells (Annexin V^−^/PI^−^, green gate), apoptotic cells (Annexin V^+^, PI^−^, red gate) and dead cells (Annexin V^+^/PI^+^, black gate) are represented for each conditions. **c.** After the experiment described in A., cells were counted using trepan blue. Histograms represent the ratio of living cells when treated with chemotherapy (100 μM carboplatin, 0.1 μM Taxol) divided by the number of living untreated cells. Mean (±SEM) of 3 different experiments. *p* < 0.05 (*), *p* < 0.01 (**) or *p* < 0.001 (***)

In a previous study, we demonstrated that mesenchymal cell-to-cell interaction with ovarian cancer cells enhanced pro-metastatic traits by increasing malignant cell adherence, invasion, proliferation and chemoresistance [11]. To assess the role of secreted factors, OCC and MSC extracted from ascites were seeded on transwell (Trans) on day − 1 (D-1) and co-cultured in a 1:1 ratio on D0. On day 2, OCC:MSC co-cultures were challenged with Carboplatin (Carbo) alone (10, 60, 100 or 200 μM) or in combination with Taxol (0.1 or 1 μM) for 24 h, on D3 we evaluated the resistance to chemotherapy by flow cytometry using annexin V/propidiun iodide (PI) staining (Fig. 1a). At the highest concentrations tested (200/1 μM Carbo/Taxol), the MSC displayed a survival rate of 83.3 ± 4.2% compared to 97.3 ± 1.2% in the control or 94.1 ± 3.1% and 95.5 ± 4.4% in Carbo 200 μM alone or Carbo/Taxol 200/0.1 μM (Fig. 1b). We then evaluated the effect of MSC on the survival of three ovarian cancer cells lines (Ovcar3, APOCC and Skov3) in a transwell setting (Trans), by counting the live cells after chemotherapy challenge (Fig. 1c). The highest chemoresistance was obtained for a concentration of 100/0.1 μM Carbo/Taxol with 87.8 ± 3.4% of cells alive in MSC Trans compared to 47.9 ± 2.6% without MSC for Skov3, 88.4 ± 1.9% compared to 45.9 ± 0.8% for APOCC and 92.2 ± 1.7% compared to 53.6 ± 3.0% for Ovcar3. Overall, when exposed to soluble factors secreted by MSCs, all three ovarian cancer cell lines tested exhibited a greater resistance to chemotherapy (Fig. 1c).

**Fig. 2:**
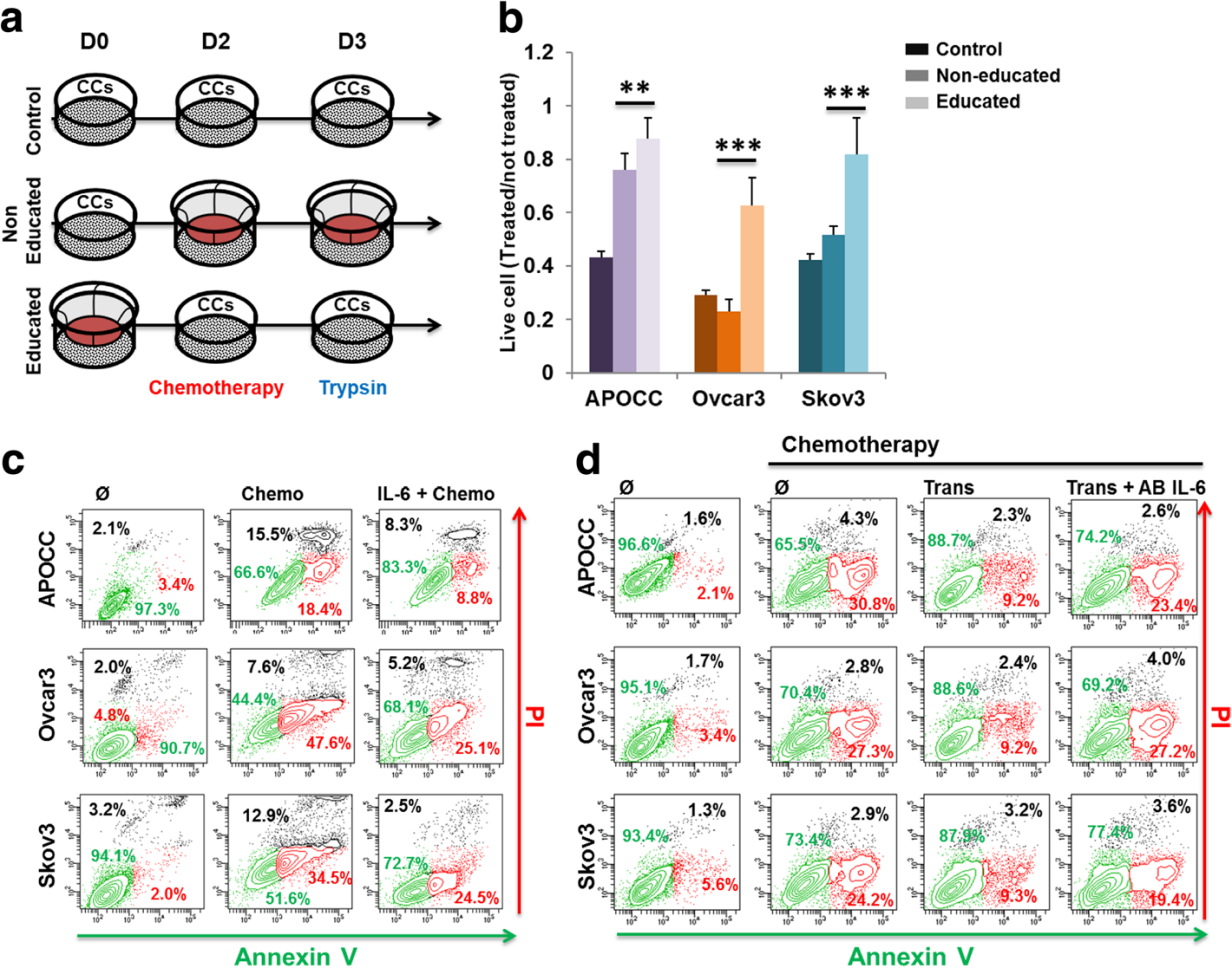
**a**. At D-1, MSC are plated on a transwell and OCCs on a well itself. At D0, MSC and OCCs are put in contact (control and removed) or not (Ø incubation) with the OCCs. At D2, MSC are kept on OCCs (control), or added to OCCs (Ø incubation) or removed from OCCs (removed) and the chemotherapy (100 μM carboplatin, 0.1 μM Taxol) is added for 24 h. At D3, cells were harvested. **b**. OCCs were treated with Carboplatin (100 μM) and Taxol (0.1 μM) for 24 h. Prior to the treatment OCCs were either culture alone (control) or 48 h with MSC (Removed). At the moment of the chemotherapy treatment MSC were removed or added to assess the role of the MSC incubation (Ø incubation). Histograms represent the percentage of living cells compared to the untreated cells. Mean (±SEM) of 3 different experiments. *p* < 0.05 (*), *p* < 0.01 (**) or *p* < 0.001 (***). **c**. OCCs were incubated or not with IL-6 (50 ng/ml) for 48 h following by chemotherapy treatment of Carboplatin (100 μM) and Taxol (0.1 μM) for 24 h. OCCs were harvested and stained with PI and Annexin V. The gates had been drawn as in B for each conditions on the plot. **d**. OCCs alone or in transwell with MSC (Trans) were incubated or not with a blocking antibody against IL-6 (20 μg/mL) for 48 h following by chemotherapy treatment of Carboplatin (200 μM) and Taxol (0.1 μM) for 24 h. OCCs were harvested and stained with PI and Annexin V. The gates had been drawn as in B for each conditions on the plot. *p* < 0.05 (*), *p* < 0.01 (**) or *p* < 0.001 (***)

We then studied how the factor secreted by MSC instruct the ovarian cancer cells to resist to chemotherapy. We either used the protocol in Fig. 1a by adding MSCs at the moment of the chemotherapy treatment (“non-educated” OCC) or we educated the OCCs for 48 h in the presence of secreted factors by MSC prior to chemotherapy (“educated” OCC) (Fig. 2a). Interestingly, educated OCCs withstood the chemotherapy challenge far better than the non-educated (ratio of 0.9 ± 0.08 vs 0.7 ± 0.06, 0.6 ± 0.1 vs 0.2 ± 0.04 and 0.8 ± 0.1 vs. 0.5 ± 0.03 cell alive (divided by the respective condition without chemotherapy) for APOCC, Ovcar3 and Skov3, respectively; Fig. 2b). Our findings suggest that education by secreted micro-environmental cues is able to elicit chemo-resistance to standard therapy.

In continuity with our previous publication [12], we decided to investigate the role of IL-6 in the chemoresistance induced by the MSC. First, we stimulated OCCs with human recombinant IL-6 (50 ng/ml) for 48 h prior the treatment with chemotherapy (100/0.1 μM carbo/Taxol). IL-6 was able to reduce the cell death caused by the chemotherapy, suggesting that IL-6 can induce a gain of chemoresistance into our three cell lines (Fig. 2c). Then we repeated our protocol of transwell co-culture (Trans) (Fig. 1a) using a blocking antibody against IL-6 (20 μg/mL). Treatment with the anti-IL-6 antibody alone was not toxic for OCCs or MSCs (Additional file 1: Figure S2). IL-6 inhibition prevented from MSC-induced chemoresistance (Fig. 2d). Similar results were found using an anti-IL6-R blocking antibody (data not shown).

### MSC induce chemoresistance in vivo

**Fig. 3:**
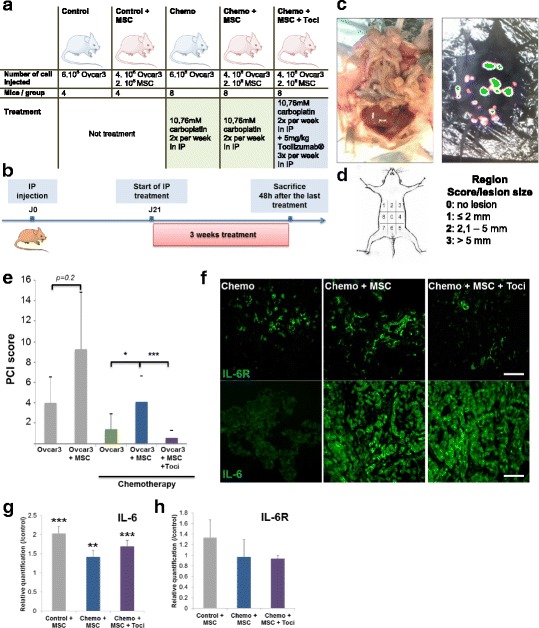
**a**. The table displays the number and type of cell injected, the number of mice and treatment for each group used for the xenograph experiment. **b**. Schematic representation of xenograph experiment time line. **c**. Representative picture of the ovarian peritoneal carcinosis seen after sacrifice of the mice (left picture). Representative picture of the bioluminescence signal (right picture). **d**. Explanation of the peritoneal carcinosis scoring. **e**. The histogram represent the mean of PCI score observed in each mouse of sacrifice. **f**. Tumors were snap-frozen after isolation and then were sectioned to 10 μm for immuno-staining. Slides were stained with Anti Human IL6-R and IL-6 antibodies and Immunofluorescence images were acquired in confocal microscopy. Scale: 100 μm. **g**-**h**. RNA was extracted from isolated tumor of each group. The relative quantification of IL-6 (**g**) and IL-6R (**h**) gene was performed by RT-PCR. The histogram represents ratios between each condition groups and the control group of their 2^–ΔΔCp^ real-time PCR values. *p* < 0.05 (*), *p* < 0.01 (**) or *p* < 0.001 (***)

To validate that factors secreted by MSCs were able to educate OCCs to resist to classical chemotherapeutic agents, we developed an in vivo ovarian peritoneal carcinosis model in immuno-compromised mice. We transduced our Ovcar3 cancer cells with a luciferase-expressing lentivirus. To avoid any bias, mice were injected with a constant number of cells (6.10^5^) even in the case of co-injection with human MSCs (Fig. 3a). Two control groups without any treatment were established (control and control +MSC) to assess the role of MSC in tumor growth. The three treated groups were designed to determine the role of MSC and IL-6 in the resistance to chemotherapy (Chemo, Chemo+MSC and Chemo+MSC + Toci). Cells were injected intra-peritonealy (IP) at day 0 and three weeks after xenograft, mice received the treatment. We treated mice with IP injections of chemotherapy twice a week with or without IP injection of Tocilizumab® trice a week (Fig. 3a-b). The distribution of the nodules was very heterogeneous and disseminated, closely mimicking human ovarian peritoneal carcinosis (Fig. 3c). The same heterogeneity and dissemination could be observed from the bioluminescence signal acquisition. Therefore, we used bioluminescence optical imaging to optimize the peritoneal carcinosis index scoring at sacrifice (PCI, Fig. 3d). We could check that the nodules detected at naked eyes had a bioluminescence signal and sometimes increase the number of nodules not detected at naked eyes.

One mouse of the “Chemo+MSC + Toci” group was excluded because no signal was detected by bioluminescence after cells injection. Without chemotherapy, we observed an increased tumoral growth in the control+MSC group compared to the control group, which wasn’t statistically significant (*p* = 0.2, Fig. 3e). However, it is important to notice that in this group, the number of tumoral cells injected was one third lower compared to the Ovcar3 alone group (4.10^5^ in control+MSC compared to 6.10^5^ in control). This demonstrates that MSCs are able to increase tumoral growth in vivo. The PCI score of Ovcar3 + MSC under chemotherapy treatment was similar to the PCI of Ovcar3 without treatment and significantly higher than the Ovcar3 alone under chemotherapy (*p* < 0.01). This result demonstrates that the presence of MSCs within tumoral nodes induce chemoresistance in co-injected mice. Conversely, when tocilizumab was added to chemotherapy in the co-injected group, the PCI significantly decreased compared with chemotherapy alone (*p* < 0.01). This confirms that the chemoresistance induced by MSC is depending on IL-6. Moreover, the complete response rate in the Tocilizumab group was higher (57.1%) than the chemotherapy alone group (12.5%), but did not reach statistically signification (*p* = 0.12). No major toxicity was observed in the Tocilizumab group: any death, infection, dehydration or diarrhea.

Immunohistochemistry on tumor sections with a human vimentin antibody confirmed the presence of human MSCs within peritoneal tumoral nodes (Additional file 1: Figure S3A). Confocal images of tumor sections stained with human Epcam antibody confirmed the engraftment of the Ovcar3 (already proved by the bioluminescence signal obtained, Additional file 1: Figure S3B). Confocal images of tumor sections demonstrated that there was no difference in IL-6 receptor staining between the 3 treated groups but that there was a more intense signal of IL-6 in the group co-injected with MSCs (Fig. 3f). This result and previous in vitro experiments confirmed that MSCs don’t induce an increase of IL-6R but an increase of IL-6 itself. Moreover, the IL-6R was still expressed after 3 weeks tocilizumab treatment, in favor of its prolonged use. We then analyzed tumor samples to decipher underlying biological mechanisms. RT-PCR of the different groups showed that MSC co-injection increased IL6 in OCCs even under Tocilizumab® treatment compared to the control (Fold 2.1 in control with MSC, 1.4 in chemo+MSC and 1.7 in Chemo+MSC + Toci, Fig. 3g). The IL6R expression remained stable upon MSC injection as well chemotherapy and anti-IL-6R therapy (Fig. 3h).

### CCL2 and CCL5 release by MSC induce OCCs IL-6 secretion and chemoresistance

**Fig. 4:**
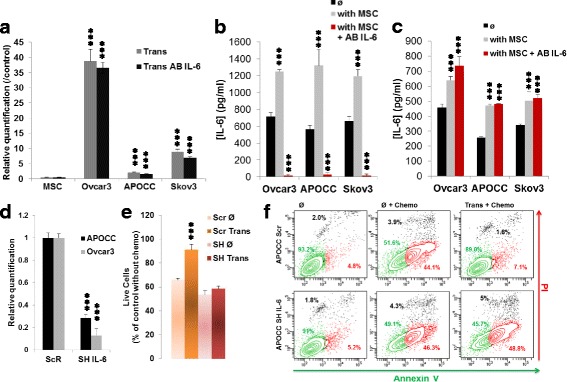
**a**. Real-time qPCR were performed on OCCs trans, OCCs trans + blocking antibody against IL-6 (20 μg/mL) and the MSC after the chemotherapy treatment (100 μM carboplatin, 0.1 μM Taxol). Relative transcript levels are represented as the ratios between the populations of interest and the control (OCCs alone with chemotherapy) of their 2^–ΔΔCp^ real-time PCR values. **b**. OCCs were incubated or not with MSC in presence or not of a blocking antibody against IL-6 (20 μg/mL) for 48 h. Elisa assay for IL-6 was performed on the supernatant of the cells. Histograms represent the mean (±SEM) for triplicates. **c**. OCCs were incubated or not with MSC in presence or not of a blocking antibody against IL-6 (20 μg/mL) for 48 h. The supernatants were removed and replace with a fresh one. After 6 h the supernatants were harvested and an Elisa assay for IL-6 was performed on the supernatant of the cells. Histograms represent the mean (±SEM) for triplicates. **d**. The relative quantification of IL-6 gene was performed by RT-PCR on APOCC and Ovcar3 after treatment with ShRNA for IL-6 or the ShRNA scramble (ScR). The histogram represents ratios between the APOCC SH-IL6 and APOCC ScR of their 2^–ΔΔCp^ real-time PCR values. **e**. Ovcar3-Scr (Scr) and Ovcar3 SH-IL6 (SH) alone (Ø) or in transwell with MSC (Trans) were treated with Carboplatin (100 μM) and Taxol (0.1 μM) for 24 h. Cells were harvested and counted. Histograms represent the percentage of living cells compared to the untreated cells. The results are presented as the mean (±SEM) of 3 different experiments. **f**. APOCC-Scr and APOCC SH-IL6 alone or in transwell with MSC (Trans) were treated with Carboplatin (100 μM) and Taxol (0.1 μM) for 24 h. Cells were harvested and stained with PI and Annexin V. The percentage of live cells (Annexin V^−^/PI^−^, green gate), apoptotic cells (Annexin V^+^, PI^−^, red gate) and dead cells (Annexin V^+^/PI^+^, black gate) are represented for each conditions on the plot. *p* < 0.05 (*), *p* < 0.01 (**) or *p* < 0.001 (***)

To investigate the origin of IL-6 secretion, we co-incubated OCCs and MSC in transwell for 48 h in absence or presence of blocking antibody against IL-6 (20 μg/mL; Trans AB IL-6) and investigated IL-6 expression in both cell types by PCR (Fig. 4a). The IL-6 expression is decreasing in MSC after the co-culture with OCCs (fold 0.5 ± 0.05). Interestingly, the IL-6 expression is significantly increased in OCCs after 48 h of incubation with MSC (fold of 38.8 ± 0.4 for Ovcar3, 2.1 ± 0.1 for APOCC and 8.9 ± 0.1 for Skov3). AB IL-6 di not significantly abolish OCCs increased expression of IL-6 after co-incubation with MSC. It implies that the IL-6 is secreted by the OCC under the influence of MSC. To verify this hypothesis, we measured the IL-6 concentration by ELISA (Fig. 4b). We confirmed that the concentration of AB IL-6 that we used was sufficient to block the secreted IL-6 in the media (18.4 ± 12.5 pg/ml compared to 717.7 ± 43.8 pg/ml in control for Ovcar3, 27.1 ± 26.5 pg/ml compared to 565.6 ± 44.9 pg/ml in control for APOCC and 18.0 ± 14.6 pg/ml compared to 665.3 ± 53.9 pg/ml in control for Skov3). Moreover, the concentration of IL-6 was significantly increased in the supernatant in presence of MSC (1252 ± 22.6 pg/ml for Ovcar3, 1324 ± 183.9 pg/ml for APOCC and 1196 ± 77.7 pg/ml for Skov3). To confirm that the increased secretion of IL-6 comes from the OCCs, we removed the MSC after 48 h of incubation with OCCs, washed 3 times with PBS and added a fresh new media on the OCCs. After 6 h the supernatant was collected and an ELISA for IL-6 was performed (Fig. 4c). IL-6 concentration is increased in the cells pre-incubated with MSC (640.4 ± 25.2 pg/ml compared to 458.0 ± 21.3 pg/ml in control for Ovcar3, 470.3 ± 8.7 pg/ml compared to 250.1 ± 7.9 pg/ml in control for APOCC and 504.4 ± 56.7 pg/ml compared to 339.6 ± 6.8 pg/ml in control for Skov3). This result explain the observation made in Fig. 1e, OCCs have an increased secretion of IL-6 upon education by MSC secreted factors. Interestingly, when OCCs are co-cultured with MSC in presence of IL-6 AB, they are still displaying an increase secretion of IL-6. Therefore, we hypothesized that the MSC secreted a factor (not IL-6) that will upregulate OCC-IL-6 secretion, protecting them from the chemotherapy.

To confirm our hypothesis, we first designed a short-hairpin RNA targeting IL-6 transcript (SH-IL-6, here after) and silenced IL6 expression in OCCs (Fig. 4d). We co-incubated MSC and OCCs (scramble, Scr or sh-IL-6, SH) for 48 h and we evaluated the expression of IL-6 by PCR in MSC and in Ovcar3 (Additional file 1: Figure S4A). We used as control cells that were not co-incubated or transfected, while IL-6 expression is significantly increased in the OCCs Scr after the co-incubation with MSC, it was not in the OCCs sh-IL-6. IL-6 expression in MSC was stable. We then treated the Ovcar3 Scr or sh-IL-6 with chemotherapy (100/0.1 μM carbo/Taxol) for 24 h after 48 h co-incubation with MSC in transwell or not and counted the cell with a hemocytometer at the end of the treatment (Fig. 4e). Silencing IL-6 in the OCCs abolished the survival to chemotherapy induced by the MSC (91.2 ± 4.5% live cell in Ovcar3 Scr Trans compared to 58.5 ± 2.4% in Ovcar3 SH Trans). Knocking-down IL-6 in the OCCs abolished the survival to chemotherapy induced by the MSC (Fig. 4f). This last result confirms that MSCs increase IL-6 production in OCCs, resulting in OCC chemoresistance.

**Fig. 5:**
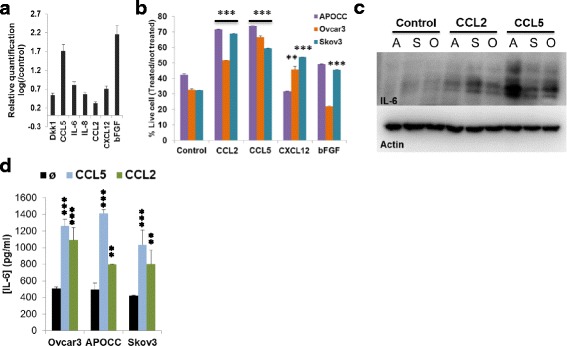
**a**. Real-time qPCR were performed on MSC before or after the experiment described in Fig. 1d. Relative transcript levels are represented as the log_10_ of ratios between the MSC after and before experiment of their 2^–ΔΔCp^ real-time PCR values. **b**. OCCs (APOCC, Ovcar3 or Skov3) were treated or not with MCP-1 (10 nM), CCL5 (100 ng/ml), CXCL12 (100 ng/ml), bFGF (10 ng/ml) for 48 h prior treatment with Carboplatin (100 μM) and Taxol (0.1 μM) for 24 h. Histograms represent the percentage of living cells compared to the untreated cells. The results are presented as the mean (±SEM) of 3 different experiments. **c**. Western blot for IL-6 was performed on OCCs (APOCC, A; Ovcar3, O; or Skov3, S) treated or not with MCP-1 (10 nM) and CCL5 (100 ng/ml) for 48 h. **d**. Ovcar3, APOCC and Skov3 were treated or not with MCP-1 (10 nM) and CCL5 (100 ng/ml) for 48 h. The supernatants were harvested and an Elisa assay for IL-6 was performed on the supernatant of the cells. Histograms represent the mean (±SEM) for triplicates. *p* < 0.05 (*), *p* < 0.01 (**) or *p* < 0.001 (***)

We performed a human cytokine array on the supernatant of MSC (starved for 24 h) to identity the cytokines released by MSC (Additional file 1: Figure S4B). MSC are releasing an important number of different cytokines (Additional file 1: Figure S4C). To narrow down our number of targets, we performed PCR on MSC before and after 48 h of contact independent co-incubation with OCCs. On Fig. 5a, we show that bFGF, CXCL12, MCP-1, IL-8, IL-6, IL-1β, CCL5, Dkk1 are up regulated after co-incubation. Then, we treated OCCs with these cytokines (100 ng/ml) for 48 h prior a chemotherapy treatment (Additional file 1: Figure S5). CCL5, CXCL12, MCP-1 and bFGF were the one inducing the highest survival apart of IL-6 itself. Increase of chemoresistance was significative (*p* < 0.001) for all the cells lines for the cytokines MCP-1 and CCL5, for Skov3 and Ovcar3 only with CXCL12 and for Skov3 with bFGF (Fig. 5b). Then, we treated OCCs for 48 h with 100 ng/ml of CCL5 and MCP-1 and investigated IL-6 expression by western blot and demonstrated an increase of IL-6 after treatment with these two cytokines (Fig. 5c). To confirm this result, we used an ELISA to measure the concentration of IL-6 after treatment with 100 ng/ml of CCL5 and MCP-1 (Fig. 5d). CCL5 was able to increase more IL-6 secretion by OCCs than MCP-1 (1260.6 ± 82.1 pg/ml compared to 1091.9 ± 149.0 pg/ml for Ovcar3, 1411.5 ± 82.7 pg/ml compared to 797.3 ± 1.4 for APOCC and 1036.9 ± 172 pg/ml compared to 802.8 ± 166.5 pg/ml for Skov3). Nevertheless, both MCP-1 and CCL5 were able to increase significantly IL-6 secretion by OCCs compared to the control (508.1 ± 15.7 pg/ml for Ovcar3, 493.3 ± 82.7 pg/ml for APOCC and 420.6 ± 7.9 pg/ml for Skov3). Our results suggest that IL-6 secretion by OCC was triggered MSC secretion of CCL5 and MCP-1 upon co-culture.

### Chemoresistance induced by MSC is dependent on phosphorylation of PYK2

**Fig. 6:**
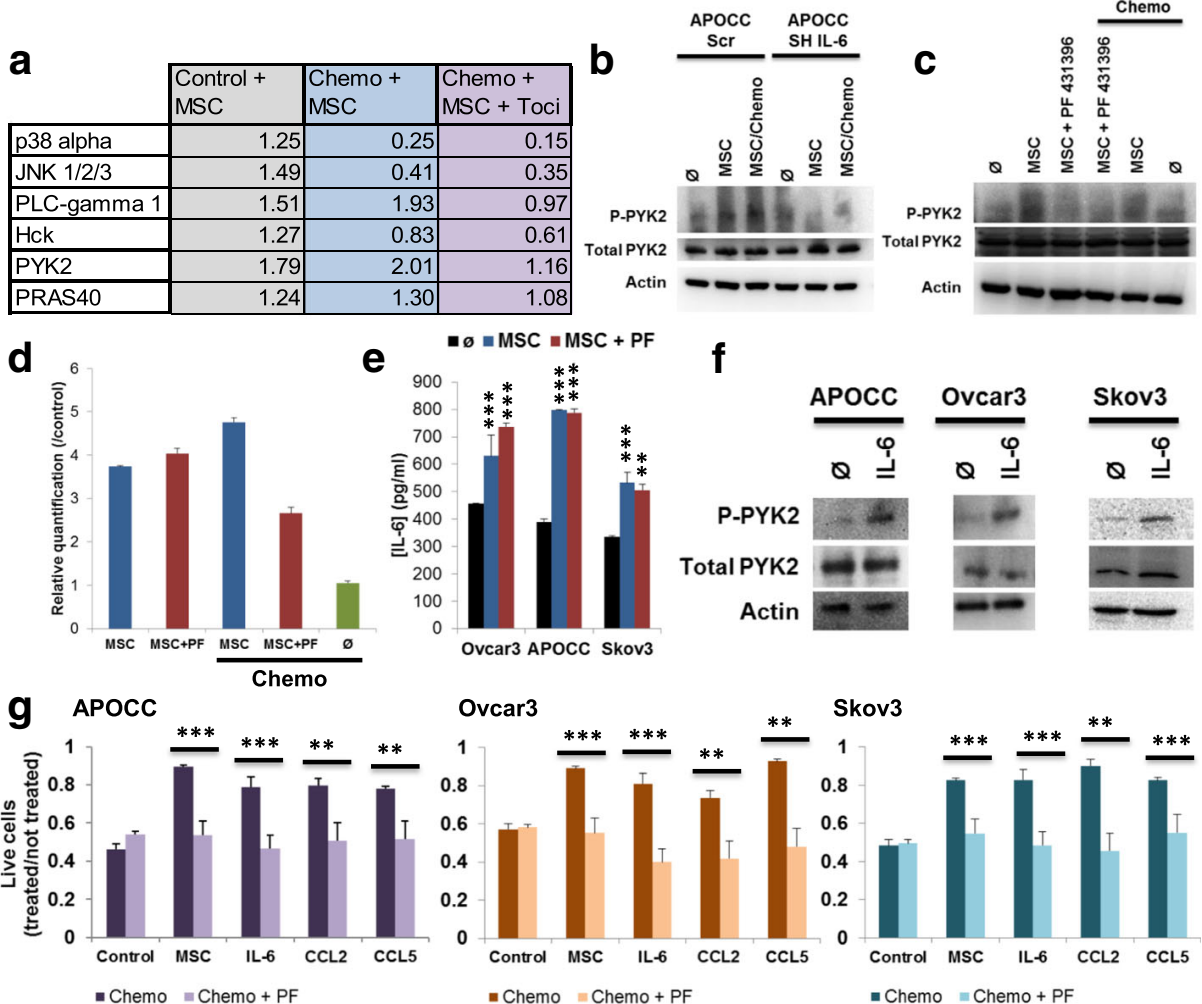
**a**. Proteins were extracted from isolated tumor of each group. Proteome profiler human phosphokinase array was performed. The table represents the fold increase of pixel density of each condition compared to control. **b**. Western blot for phospho-PYK2 and total-PYK2 was performed on OCCs (APOCC and APOCC SH-IL6) co-incubated or not with MSC for 48 h and treated or not with chemotherapy Carboplatin (100 μM) and Taxol (0.1 μM) for 24 h. **c**. Western blot for phospho-PYK2 and total-PYK2 was performed on OCCs (APOCC) co-incubated or not with MSC in presence or not of PF431396 (5 μM) for 48 h and treated or not with chemotherapy Carboplatin (100 μM) and Taxol (0.1 μM) for 24 h. **d**. The relative quantification of IL-6 (gene was performed by RT-PCR. The histogram represents ratios between each condition and the control group (no MSC, no chemotherapy) of their 2^–ΔΔCp^ real-time PCR values. **e**. OCCs (Ovcar3, APOCC and Skov3) were co-incubated or not with MSC in presence or not of PF431396 (5 μM) for 48 h. Supernatants were harvested and an Elisa assay for IL-6 was performed on the supernatant of the cells. Histograms represent the mean (±SEM) for triplicates. **f**. Western blot for phospho-PYK2 and total PYK2 was performed on OCCs (Ovcar3, APOCC and Skov3) incubated with IL-6 (50 ng/ml) for 6 h. **g**. OCCs (Ovcar3, APOCC and Skov3) were treated with MCP-1 (10 nM), CCL5 (100 ng/ml), IL-6 (50 ng/ml) or co-incubated with MSC, in presence (light graph) or not (plain graph) of PF431396 (5 μM) for 48 h prior treatment with Carboplatin (100 μM) and Taxol (0.1 μM) for 24 h. Histograms represent the ratio of living cells compared to the not treated cells. *p* < 0.05 (*), *p* < 0.01 (**) or *p* < 0.001 (***)

To understand how OCCs respond to MSC, we investigated the phosphorylation profiles of kinases and their protein substrates. We used a Human Phospho-Kinase Array to detect the relative site-specific phosphorylation of 43 kinases and 2 related total proteins on tumors from 4 different groups of mice (control, MSC, MSC+ chemo, MSC + chemo + Toci; Additional file 1: Figure S6A). Phosphorylation state for each kinase was evaluated by measuring the pixel density of each dot plots. The results are represented as fold increase compared to control group. Kinases that were significantly up-regulated with MSC and down-regulated with Tocilizumab are represented in Fig. 6a. PLC-ɣ1, PYK2 and PRAS40 are the only kinase that are increased in OCCs with MSC co-injection even under chemotherapy and decreased by Tocilizumab. To confirm the role of PLC-ɣ1, PYK2 or PRAS40 in MSC induced chemoresistance, we performed a phosphokinase array on APOCC and APOCC SH-IL6 co-incubated or not with MSC and treated or not with chemotherapy (Additional file 1: Figure S6B). More kinases were phosphorylated in APOCC co-incubated with MSC and not in APOCC sh-IL6 compared to their respective control (Additional file 1: Figure S6C). PYK2 appears to be the more phosphorylated under MSC co-incubation (fold 8.89 compared to APOCC), moreover its phosphorylation state is basal in the case of APOCC sh-IL6 co-incubated with MSC (fold 1.12 compared to APOCC sh-IL6). To confirm the phosphorylation of PYK2 under MSC co-incubation we performed a western blot (Fig. 6b). PYK2 is phosphorylated in APOCC co-incubated with MSC but not in APOCC sh-IL6. This result suggests that the PYK2 could be phosphorylated by APOCC IL-6 secretion induced by MSC. PF-431396 is a potent inhibitor of PYK2 and FAK. When using PF-431396 (5 μM) during the co-incubation of APOCC with MSC, PYK2 phosphorylation is abolished (Fig. 6c). We investigated the expression of IL-6 by APOCC under PF-431396 treatment by PCR (Fig. 6d). PF-431396 didn’t affect the expression of IL-6 by APOCC alone. We then used an Elisa array to evaluate the concentration of IL-6 in the supernatant to see if a treatment with PF-431396 (5 μM) was able to abolish the IL-6 secretion by OCCs (Fig. 6e). PF-431396 was not able to abolish the increase of IL-6 secretion by OCCs under MSC co-culture (737.5 ± 13.5 pg/ml under PF-431396 compared to 628.8 ± 75.7 pg/ml without PF-431396 and 435.0 ± 2.9 pg/ml in control for Ovcar3, 788.1 ± 14.5 pg/ml under PF-431396 compared to 797.4 ± 1.4 pg/ml without PF-431396 and 387.9 ± 12.2 pg/ml in control for APOCC and 505.3 ± 20.6 pg/ml under PF-431396 compared to 532.7 ± 36.6 pg/ml without PF-431396 and 335.3 ± 3.3 pg/ml in control for Skov3). Then we treated the 3 OCCs with IL-6 (50 ng/ml) for 6 h and performed a western blot of Phospho-PYK2 (P-PYK2) and total PYK2 (Fig. 6f). PYK2 was phosphorylated under IL-6 treatment in the three cell lines. Finally, we assessed the resistance to chemotherapy in different condition under PF-431396 treatment. Interestingly, PF-431396 is able to abrogate the resistance in all conditions (Fig. 6g).

**Fig. 7:**
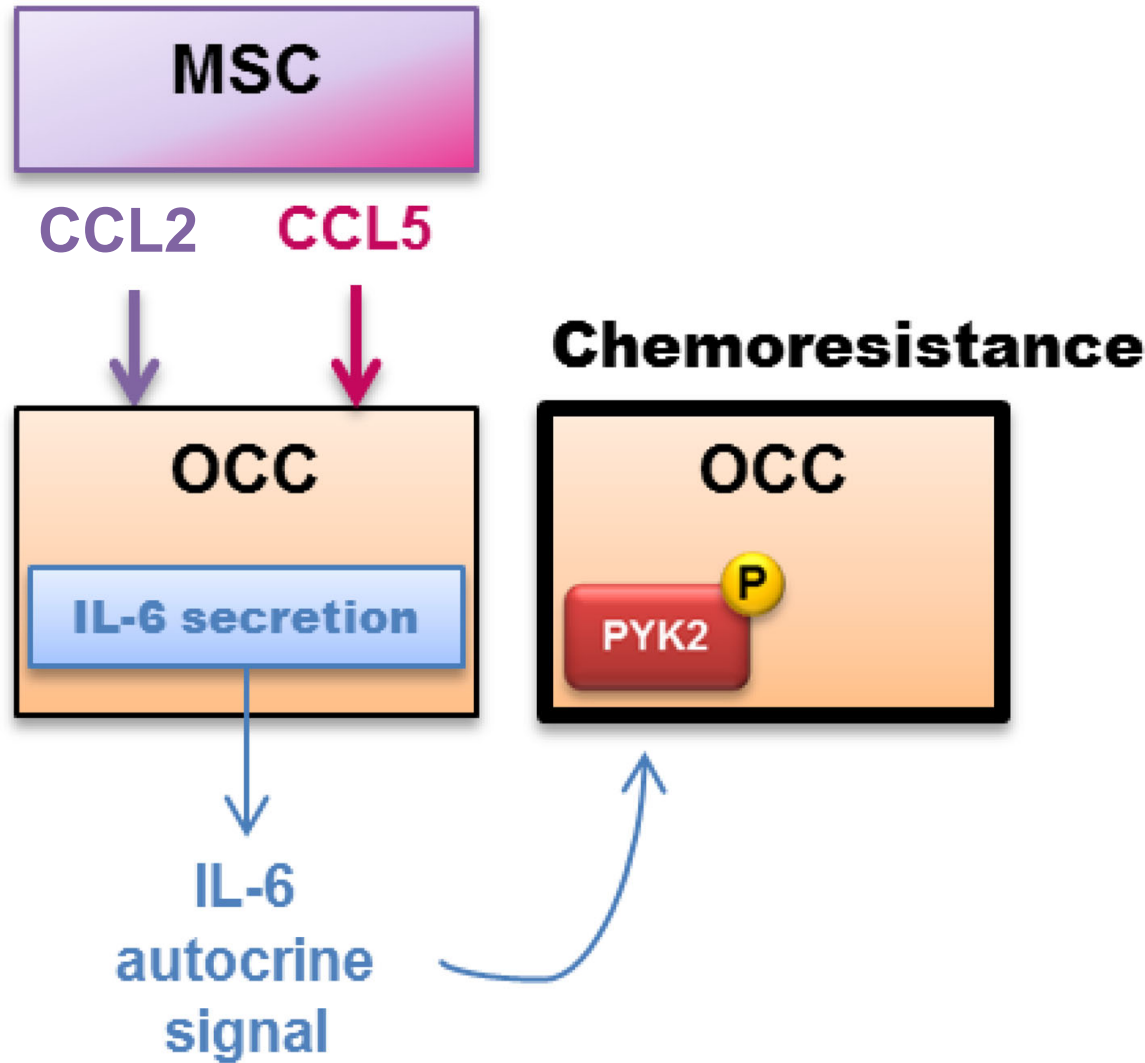
Schematic representation of MSC role in OCC chemoresistance

Altogether, our findings suggest a phosphorylation of PYK2 after IL6 OCC auto-stimulation upon co-culture with MSC resulting in OCC chemoresistance (Fig. 7).

## Discussion

We demonstrated that MSCs are found in patients’ ascites and are able to induced OCC chemoresistance in vitro. We showed an IL-6 dependent induced OCC chemoresistance in OCC upon MSC co-culture both in vitro and in vivo, reversed by the use of tocilizumab, an anti-IL6R antibody*.* Through secretion of CCL2 and CCL5, MSCs are able to induce IL-6 production in OCCs. IL-6 will have an autocrine effect on OCCs themselves and induce the phosphorylation of PYK2 leading to chemoresistance.

Previous report showed that MSCs (CD44+, CD73+, CD90+) represent around 6% of the full cell population in human ovarian tumor ascites [21]. Another team demonstrated that ascites-derived stromal cells, (also called Carcinoma-associated mesenchymal stromal cells and hospicell) could be isolated from ascites of patients with ovarian carcinosis and participated to tumorigenicity, chemoresistance, metastasis and angiogenesis in ovarian cancer [19, 22, 23].

MSC has already been associated with increased resistance to treatment upon contact [13]. Here, we focused on contact-free induction of chemoresistance. For the first time, we were able to establish that MSC induced an autocrine regulation of chemoresistance in OCC. In fact, while MSC-CCL2 and MSC-CCL5 are known involved in resistance to chemotherapy [24–26], here we showed that they are just having an indirect role by inducing the expression of IL-6 in OCC. These three cytokines have been shown to be intimately related in cardiac fibroblast [27], endometrial stromal fibroblasts [28] as well as in cancer associated MSC [29, 30]. Nevertheless, while IL-6 is known to induce the expression of CCL2 and CCL5 [27, 30–32], to our current knowledge, we are the first to report that CCL2 and CCL5 can induce IL-6 expression.

IL6 is an important cytokine in the ovarian cancer cytokine network [33]. Increased expression of IL6 and its specific receptor IL6Rα was even associated with disease stage [34]. Coward showed that intensity of IL-6 staining in malignant ovarian cancer cells significantly associated with poor prognosis in a series of 221 patients [35]. In vitro treatment of ovarian cancer cells with an anti-IL-6 therapy reduced tumor growththe tumor-associated macrophage infiltrate and angiogenesis. This is also concordant with a previous work showing a decreased infiltration of OCC in a 3D model using amniotic membrane to mimick peritoneal carcinosis [12]. The question of its implication in chemoresistance is therefore important. Using a blocking antibody strategy as well SH-RNAwe demonstrated that the autocrine production of IL-6 is responsible for OCC chemoresistance. Autocrine production of IL-6 has already been shown to confer cisplatin and taxol resistance in OCC. But here we demonstrated that this autocrine production was induced by the MSC themselves

To confirm the role of MSC and IL-6 in chemoresistance, we used an ovarian peritoneal carcinosis model in nude mice using bioluminescence. To evaluate the peritoneal carcinosis, we used a mouse modified “Peritoneal Carcinosis Index” (PCI). Additionally, we used bioluminescence to increase the sensitivity and specificity of this evaluation allowing finding small nodules in less accessible locations such as under diaphragmatic cupolas [36, 37]. While co-injection of MSC with OCC involved a lower number (33%) of OCCs, the resulting peritoneal carcinosis was comparable to the one in the mono-injection groups. The chemo-protection when co-injecting MSCs and OCCs was significant. Our model is original by using anti-IL-6 therapy in combination with chemotherapy rather than as a single agent as previously reported [35, 38]. Platinum based chemotherapy is the referent treatment with excellent initial response. Unfortunately, most patients will display chemoresistant recurrences and second line strategies use other single agent chemotherapy. The association between anti IL-6Ra and platinum based chemotherapy is original by targeting the interaction with cancer microenvironment. Two clinical studies using anti-IL-6 therapy reported a good tolerance and effects on microenvironment [35, 39]. Interestingly, the use of anti-IL6 in monotherapy resulted in a low response rate around 5% [35] against > 50% in association with chemotherapy [39]. The authors report an increased IL6 and sIL6 rates in patients treated by anti-IL-6 and chemotherapy, which is in accordance with our findings. We also found a stable expression of IL6R expression and increased IL-6 by IHC in samples from mice treated with Anti-IL-6 and chemotherapy. Moreover, the persistence of IL-6R expression after 3 weeks anti-IL-6 treatment confirms the rational to prolonged anti-IL-6 treatment in our model. New clinical studies should now confirm the potential benefice and determine the modalities and length of this anti-IL6 therapy.

To our knowledge, this is the first report PYK2 pathway inducing chemoresistance after IL6 stimulation in a mouse study of ovarian cancer. In 2000, a team revealed that activation of PYK2 was inhibited by IL-6 in multiple myeloma [40]. Conversely, in 2015, IL-6 was shown to be able to activated PYK2 in the context of respiratory diseases [41]. More recently, Meads and collaborator demonstrated that PYK2 is positioned upstream of JAK1/STAT3 signaling and that it is a critical mediator of a novel survival pathway activated in the context of co-stimulation of cancer cell and the microenvironment through especially IL-6 [42].

PYK2 is a member of the focal adhesion kinase (FAK) subfamily. Recently, several FAK inhibitors were shown to suppress ovarian cancer chemoresistance and enable them to respond routine chemotherapies [43–45]. In ovarian cancer context, cell growth and survival can be facilitated in a kinase-independent manner through activation of PYK2 [46]. PYK2 was also shown to be a critical downstream signaling pathway for ascites-induced cell migration [47]. Moreover, inhibition of PYK2 could enhance apoptosis in OCC [48]. This could support the clinical use of FAK/PYK2 inhibitor such as VS-6063 (DEFACTINIB) that is already in phase I trial for solid tumor [49, 50] and in phase II study for patients with KRAS mutant in non-small cell lung cancer.

## Conclusions

The involvement of MSC in development of OC resistance to chemotherapy seems to be clear, yet they are still not microenvironment targeted therapy in the standard treatment against ovarian cancer. The disruption of OCC–MSC crosstalk is now mandatory [51]. Our study reinforces the role of IL-6 in the occurence of chemoresistance in EOC. Taken altogether, our results suggest different ways of blocking the IL-6 induced chemoresistance, i) inhibited the CCL2/CCL5 activation of IL-6, ii) use direct IL-6 blocking strategy, iii) use FAK inhibitor. The development of multi-treatment modalities blocking microenvironment negative impact as well as immunotherapies in addition to classical cytotoxic molecules is now mandatory in EOC. Further clinical trials associating an anti-IL6 as a microenvironment inhibitor and chemotherapy may be conducted to improve our therapeutic strategies in EOC.

## Additional files

Additional file 1: Figure S1. A. EOC in situ surrounded by ascites. Cells Epcam− (red gate) were cell sorted and cultured. B. Phase contrast pictures of cells sorted in A. Scale bar: 50 μm C. Flow cytometry for every MSC markers on cells sorted in A. D. Cell sorted in fig. 1a were cultured for few days and stained with CD29 (green), CD105 (green), CD90 (red), CD73 (red) antibodies. Scale: 10 μm. Figure S2. OCCs were treated for 48 h with a blocking antibody against IL-6 (20 μg/mL). The percentage of live cells (green gate), apoptotic cells (red gate) and dead cells (black gate) are represented on the plot. Figure S3. A. Paraffin-embedded vimentin immunohistochemistry for the mouse group Control + MSC and Chemo + MSC. B. Confocal images for Epcam on 10μm sections of snap-frozen tumors. Scale: 100 μm. Figure S4. A. Relative quantification of IL-6 gene in RT-PCR on Ovcar3 (orange) and APOCC (purple) treated with SH IL-6 (SH) or scrambled (Scr), and MSC (grey) before (No cocu) or after co-incubation with OCCs scr or SH for 48 h. The histogram represents ratios between the transwell and the control condition of their 2–ΔΔCp real-time PCR values. B. Acquisition of the membrane in chemiluminescence. C. Hierarchical representation of the pixel density of each dot of the cytokine array. Figure S5. Phase contrast of OCCs after treatment with IL-8 (50 ng/ml), Dkk1 (20 ng/ml), IL-6 (50 ng/ml), MCP-1 (10 nM), CCL5 (100 ng/ml), CXCL12 (100 ng/ml), bFGF (10 ng/ml) for 48 h prior treatment with Carboplatin (200 μM) and Taxol (0.1 μM) for 24 h. Figure S6. A. Proteome profiler human phosphokinase array. B. Proteome profiler human phosphokinase array. C. Fold increase of pixel density of each condition compared to APOCC control (blue part) or to APOCC SH-IL6 (purple part). (PDF 1100 kb)

Additional file 2: Table S1. Primers list. (DOCX 14 kb)
