# Supplementary material for: CCL2/CCL5 secreted by the stroma induce IL-6/PYK2 dependent chemoresistance in ovarian cancer
Source: Mol Cancer. 2018 Feb 19;17:47. doi: 10.1186/s12943-018-0787-z (PMC5817856; doi:10.1186/s12943-018-0787-z)
Supplement: Supplementary file 1 — A. EOC in situ surrounded by ascites. Cells Epcam− (red gate) were cell sorted and cultured. B. Phase contrast pictures of cells sorted in A. Scale bar: 50 μm C. Flow cytometry for every MSC markers on cells sorted in A. D. Cell sorted in fig. 1a were cultured for few days and stained with CD29 (green), CD105 (green), CD90 (red), CD73 (red) antibodies. Scale: 10 μm. Figure S2. OCCs were treated for 48 h with a blocking antibody against IL-6 (20 μg/mL). The percentage of live cells (green gate), apoptotic cells (red gate) and dead cells (black gate) are represented on the plot. Figure S3. A. Paraffin-embedded vimentin immunohistochemistry for the mouse group Control + MSC and Chemo + MSC. B. Confocal images for Epcam on 10μm sections of snap-frozen tumors. Scale: 100 μm. Figure S4. A. Relative quantification of IL-6 gene in RT-PCR on Ovcar3 (orange) and APOCC (purple) treated with SH IL-6 (SH) or scrambled (Scr), and MSC (grey) before (No cocu) or after co-incubation with OCCs scr or SH for 48 h. The histogram represents ratios between the transwell and the control condition of their 2–ΔΔCp real-time PCR values. B. Acquisition of the membrane in chemiluminescence. C. Hierarchical representation of the pixel density of each dot of the cytokine array. Figure S5. Phase contrast of OCCs after treatment with IL-8 (50 ng/ml), Dkk1 (20 ng/ml), IL-6 (50 ng/ml), MCP-1 (10 nM), CCL5 (100 ng/ml), CXCL12 (100 ng/ml), bFGF (10 ng/ml) for 48 h prior treatment with Carboplatin (200 μM) and Taxol (0.1 μM) for 24 h. Figure S6. A. Proteome profiler human phosphokinase array. B. Proteome profiler human phosphokinase array. C. Fold increase of pixel density of each condition compared to APOCC control (blue part) or to APOCC SH-IL6 (purple part). (PDF 1100 kb) [file 12943_2018_787_MOESM1_ESM.pdf]

Supplementary figure 1

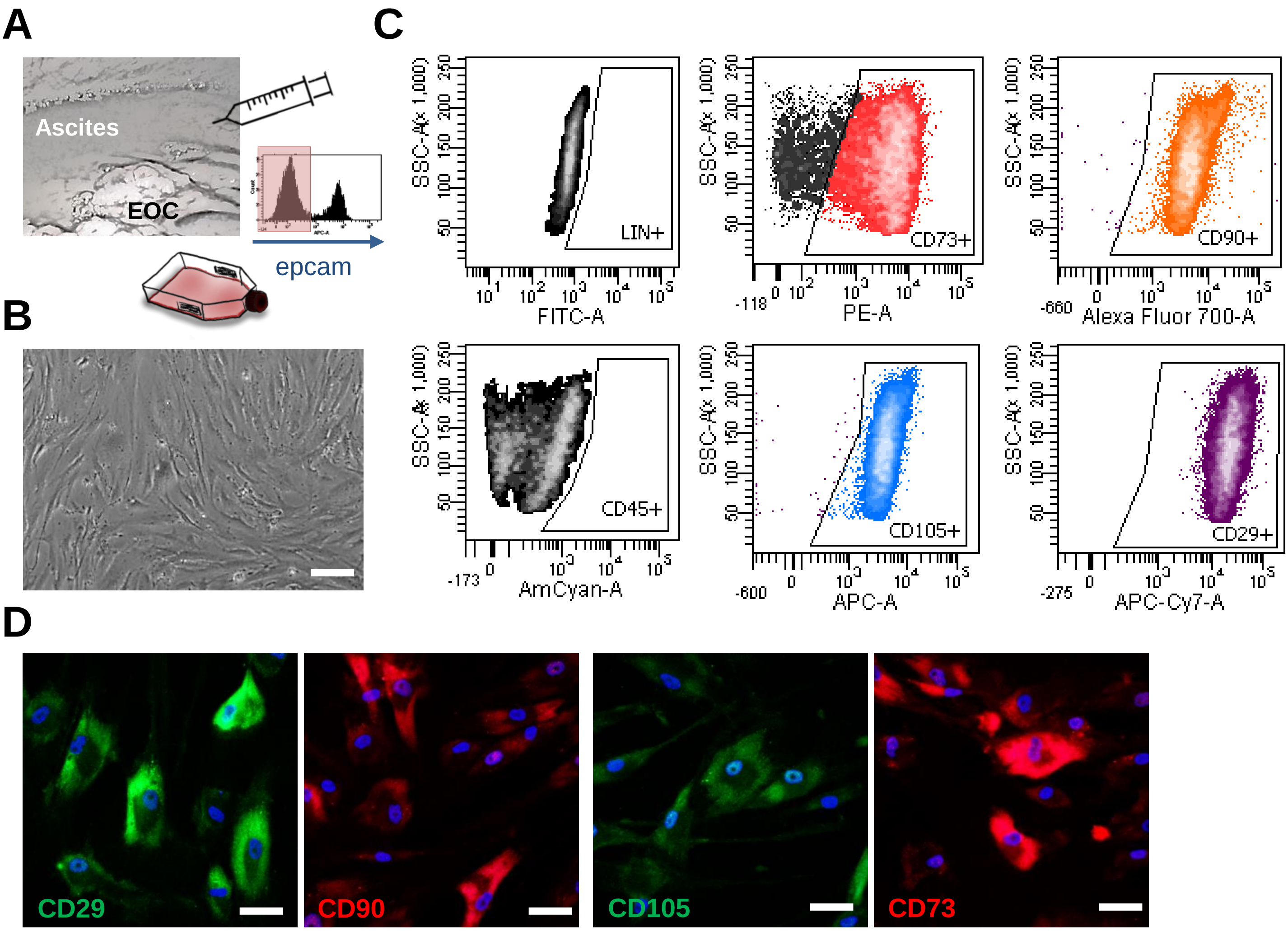

Supplementary figure 2

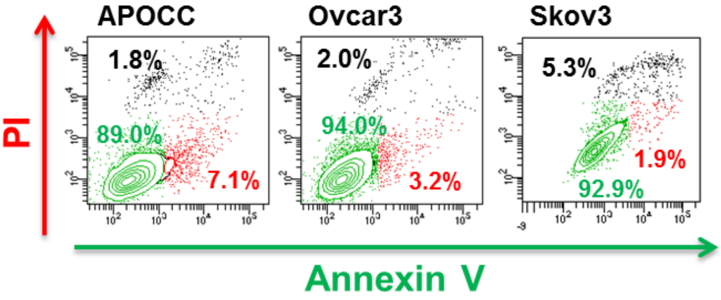

Supplementary figure 3

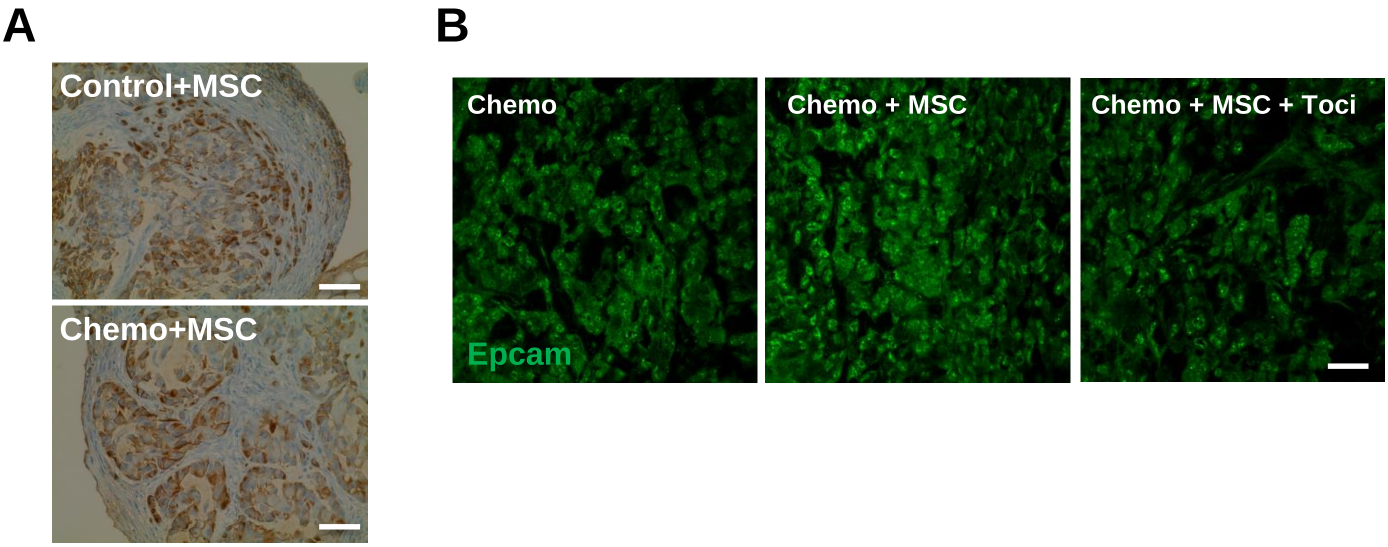

Supplementary figure 4

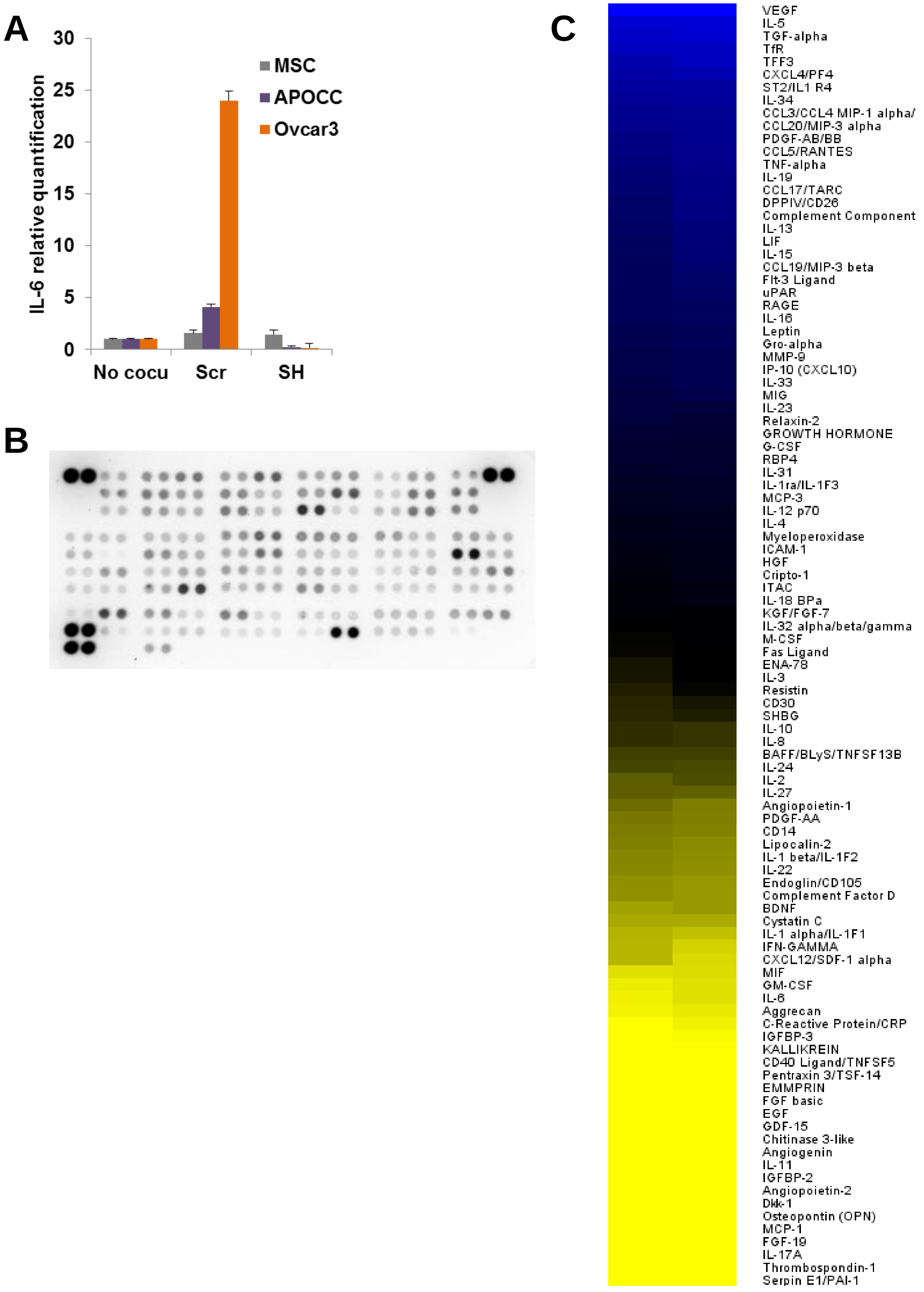

Supplementary figure 5

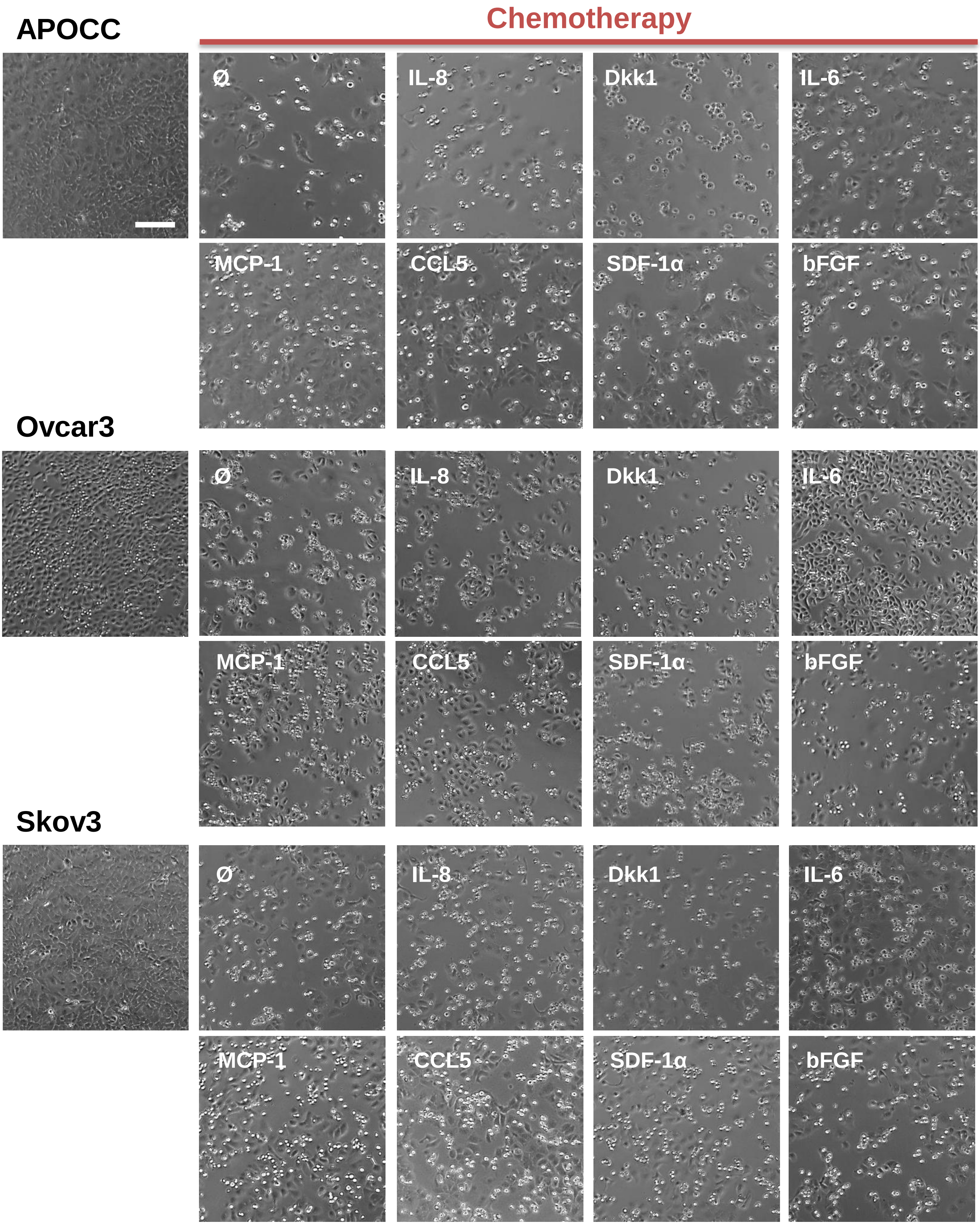

Supplementary figure 6

A

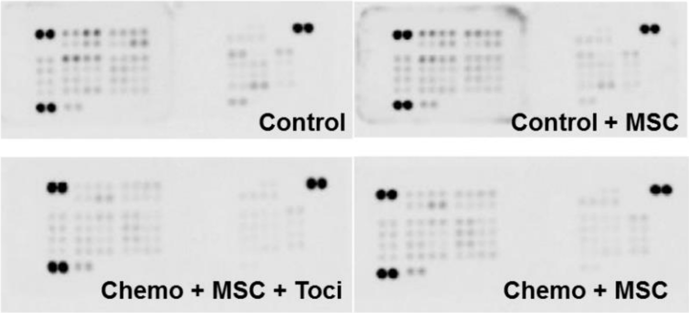

B

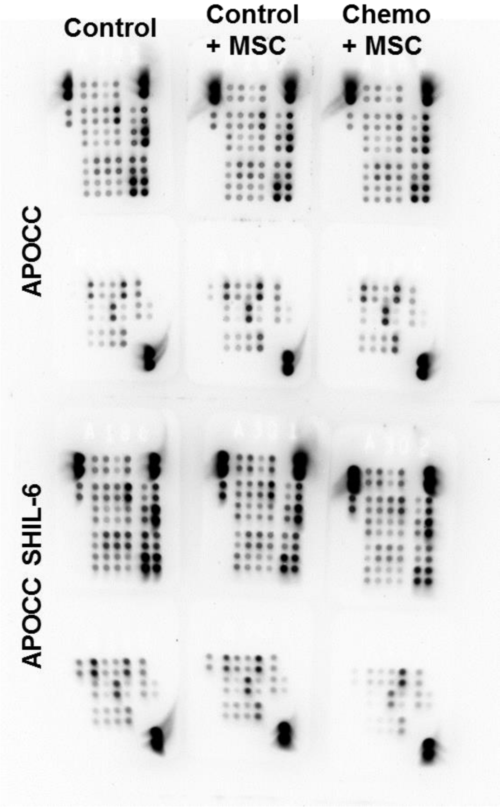

C

	APOCC MSC	APOCC MSC chemo	APOCC SH MSC	APOCC SH MSC chemo
Akt 1/2/3 (S473)	2.12	1.86	0.74	0.59
HSP27	1.20	1.19	0.80	0.64
p70 S6 Kinase	1.32	1.25	1.06	1.05
c-Jun	1.43	1.54	1.35	0.64
p70 S6 Kinase	1.33	1.01	0.94	0.50
RSK 1/2/3	1.40	1.47	0.93	0.56
Fyn	1.26	1.34	1.29	1.22
Fgr	1.28	1.11	1.03	0.96
STAT5b	1.28	1.09	0.78	0.48
STAT3 (Y705)	2.03	1.92	1.28	0.29
p27	1.31	1.06	1.09	0.19
PLC-γ1	1.49	1.30	1.43	0.18
Hck	1.29	1.33	1.26	1.25
Chk-2	1.26	1.12	0.90	0.78
FAK	1.45	0.97	1.18	1.05
PDGF Rβ	1.30	1.21	0.81	0.53
STAT5a/b	1.32	1.20	1.21	0.68
STAT3 (S727)	1.57	1.50	0.63	0.08
WNK1	2.09	1.53	0.66	0.04
PYK2	8.89	2.36	1.12	0.05
HSP60	11.34	4.28	0.64	0.04
