# Supplementary material for: CCL2/CCL5 secreted by the stroma induce IL-6/PYK2 dependent chemoresistance in ovarian cancer
Source: Mol Cancer. 2018 Feb 19;17:47. doi: 10.1186/s12943-018-0787-z (PMC5817856; doi:10.1186/s12943-018-0787-z)
Supplement: Supplementary file 2 — Primers list. (DOCX 14 kb) [file 12943_2018_787_MOESM2_ESM.docx]

| **Primer** | **Forward** | **reverse** |
| --- | --- | --- |
| **IL-1β** | CCTGTACGATCACTGAACTG | TGGGCAGACTCAAATTCCAG |
| **IL-8** | ATGACTTCCAAGCTGGCCGT | TCCTTGGCAAAACTGCACCT |
| **IL-6** | GTCAGGGGTGGTTATTGCAT | AGTGAGGAACAAGCCAGAGC |
| **IL-6R** | ACTGGTCAGCACGCCTCT | GGGACCATGGAGTGGTAGC |
| **MCP1** | ATGAAAGTCTCTGCCGCCCTCA | GAGATCTGTGCTGACCCCAA |
| **SDF1α** | AGATGCTTGACGTTGGCTCT | AAGGTCGTGGTCGTGCTG |
| **Dkk1** | CAGGATTGTGTTGTGCTAGA | TGACAAGTGTGAAGCCTAGA |
| **CCL5** | CCATGAAGGTCTCCGCGGCAC | CCTAGCTCATCTCCAAAGAG |
| **bFGF** | CACCAGGCCACTTCAAGGA | GATGGATGCGCAGGAAGAA |
| **GAPDH** | agccacatcgctcagacac | gcccaatacgaccaaatcc |
